# Dr. John Chapman

**Published:** 1894-12-08

**Authors:** 


### Obituary.

### DR. JOHN CHAPMAN.

To all who knew Dr. John Chapman, his death in Paris on November 25th, was a terrible blow. One of the handsomest, one of the most original men of the century, he was ever a prominent figure in the world of letters. There was a time when he was a prominent figure in hospital affairs, and it was the treatment he received from the hospital world which drove him from England. Born in 1821, John Chapman started in life as a bookseller in the Strand, a business which in time grew into the well-known publishing firm of Chapman Brothers, which brought out important works by Martineau and Grey, and translations of writings of Fichte, Jean Paul, Schelling, and Edgar Luinet; and in the intervals of book-selling, publishing, and writing, John Chapman took his M.D., his M.R.C.P., and his M.R.C.S. At his house there used to gather the most brilliant minds of the day. Side by side with the literary grew the medical life; Dr. John Chapman became physician to the Farringdon Dispensary and assistant physician to the Metropolitan Free Hospital. He was too active a man to wink at abuses, and what he saw of the out-patient department at the Metropolitan was more than he could stand. Dr. Chapman wrote two articles in the *Westminster Review*, pointing out the extravagant items of expenditure, the unspairing advertisements, and the extraordinary increase of gratuitous medical relief, and the then gross abuse of the out-patient department. What followed shall be told in Dr. Chapman's own words in a letter addressed to the writer of this notice: "Medical charity is a subject which, as you see from my articles, has interested me greatly. It has, in fact, cost me my position as assistant physician to the Metropolitan Free Hospital. The Board asked me to resign, the heads of the profession, especially the late Sir Thomas Watson, the late Sir W. Ferguson, Sir James Paget, and others, urged me not to resign, but to leave the onus on the hospital of expelling me. Accordingly I continued to see my patients regularly, until one morning, going to the hospital as usual, I found the door shut in my face. This fact made me become an exile, and I have since resided in Paris." It is strange that all the daily papers which gave long obituary notices of Dr. Chapman missed this, the most prominent fact in his life. As a literary man, as the editor of the *Westminster Review*, even as the physician to the English circle in Paris, they knew him; but as a hospital reformer they knew him not—so quickly has the sound of that old battle at the Metropolitan died away. It only remains to say that the views which Dr. Chapman advocated, and for which he suffered, are now generally held by all interested in hospital organisation, and were advocated strongly by the late House of Lords Commission.

